# Clinical Significance of Novel Neutrophil-Based Biomarkers in the Diagnosis and Prediction of Response to Infliximab Therapy in Crohn’s Disease

**DOI:** 10.3389/fimmu.2022.865968

**Published:** 2022-03-04

**Authors:** Zhou Zhou, Yinghui Zhang, Xue Yang, Yan Pan, Liangping Li, Caiping Gao, Chong He

**Affiliations:** ^1^ Department of Gastroenterology, Sichuan Provincial People’s Hospital, University of Electronic Science and Technology of China, Chengdu, China; ^2^ Clinical Immunology Translational Medicine Key Laboratory of Sichuan Province, Sichuan Provincial People’s Hospital, University of Electronic Science and Technology of China, Chengdu, China

**Keywords:** Crohn’s disease, biomarker, inflammatory bowel disease, neutrophil, albumin, bilirubin, neutrophil-to-albumin ratio, neutrophil-to-bilirubin ratio

## Abstract

With the increasing incidence and prevalence, Crohn’s disease (CD) has become one of the most challenging diseases in both diagnosis and treatment of gastroenterology. Evaluation of the disease activity and mucosal healing guides clinical decisions regarding subsequent therapy for CD. In this study, we enrolled a total of 144 patients with CD and 239 healthy controls were enrolled. Clinical characteristics and laboratory parameters of enrolled subjects were retrieved from the electronic medical record database of our hospital. Serum cytokine levels were measured by enzyme-linked immunosorbent assay (ELISA). Mucosa expression levels of inflammatory agents were measured by quantitative RT-PCR (qRT-PCR). We identified two neutrophil-based indexes, the neutrophil-to-albumin ratio (NAR) and neutrophil-to-bilirubin ratio (NBR), both of which had not yet been explored in CD or UC. NAR and NBR were significantly increased in patients with CD compared to those in healthy controls, and both indexes showed significantly positive correlations with CD activity and inflammatory load. In note, NAR and NBR showed better performance than blood neutrophil percentage, serum albumin, or bilirubin alone in these scenarios. More importantly, both NAR and NBR discriminated CD patients who completely or partially responded to infliximab (IFX) induction therapy from those with primary non-response. Our observations suggest that NAR and NBR may serve as promising biomarkers in the diagnosis and prediction of response to IFX therapy in CD.

## Introduction

Inflammatory bowel disease (IBD) including Crohn’s disease (CD) and ulcerative colitis (UC) is now considered as a global health issue with its increasing incidence and prevalence (1). IBD is well-known to be a refractory and recurrent immunologic disorder of the gastrointestinal tract, and it appears to result from dysregulation of the immune system. Although clinical remission has been traditionally considered as the initial goal of therapy for IBD, achievement of mucosal healing is now regarded to be the therapeutic goal with advances in methods for disease assessment (2, 3). Accumulating evidences have shown that mucosal healing in IBD patients indicates a lower hospitalization rate and improved prognosis, and higher risk of disease-related complications has been observed when colonoscopy monitoring rates are reduced (3–6). Therefore, evaluation of the disease activity and mucosal healing guides clinical decisions regarding subsequent therapy for IBD.

Biomarkers in IBD are able to help monitor disease activity in clinical practice. A biomarker is a biological observation that is able to predict a clinical outcome, which is difficult to observe directly (7). Efforts have been made to differentially diagnose IBD from functional bowel disease, monitor disease activity, and predict therapeutic effect, recurrence, prognosis by blood and stool tests, such as erythrocyte sedimentation rate (ESR), C-reactive protein (CRP) and fecal calprotectin (8–11). Aside from currently available biomarkers, most of which in fact deliver suboptimal performance, gastrointestinal endoscopy remains the most powerful tool to monitor the inflammatory activity of IBD. However, its application is limited because of its invasiveness (12). Additionally, those who undergo endoscopy have complained embarrassment, discomfort caused by bowel preparation, and increased abdominal pain (13, 14). To better manage IBD, the search for reliable and non-invasive biomarkers that can be easily accessible and cost-effective is necessary and urgent.

Although markers based on blood routine examination including white blood cell (WBC), CRP and ESR are commonly applied as inflammatory indicators in routine clinical practice for determining IBD activity, it is difficult to reflect the disease activity or predict disease progression by using a single biomarker due to their low sensitivity and specificity (15). Recent researches focusing on the combination of two parameters reinvigorate the examination of white blood cell patterns and emerging evidences have revealed the potential values of the neutrophil-to-lymphocyte ratio (NLR) (16), platelet-to-lymphocyte ratio (PLR) (17), and lymphocyte-to-monocyte ratio (LMR) (18) for disease activity assessment and therapeutic effect prediction. For example, NLR and PLR are elevated in active UC patients compared to those in inactive patients and healthy controls. In addition to blood cell tests, serum biochemistry examinations have provided several indices to assist to reflect IBD activity. Serum albumin (ALB), bilirubin (BIL), and uric acid (UA) are significantly reduced in patients with CD, especially those with severe disease activity, and lower levels of them are companied by increased inflammatory indices (19, 20). However, limited studies have paid attention to biomarkers derived from the combination of blood cell and serum biochemistry examinations.

In the current study, we combined blood neutrophils and serum ALB, BIL as the neutrophil-to-ALB ratio (NAR) and neutrophil-to-BIL ratio (NBR), respectively, and aimed to compare NAR and NBR between the control group and CD patients. Additionally, we sought to explore whether these two indexes were able to reflect the disease activity and predict response to infliximab in patients with CD.

## Materials and Methods

### Subjects

Our study was conducted in accordance with the Declaration of Helsinki and approved by the Institutional Review Board for Clinical Research of Sichuan Provincial People’s Hospital (No. 201968). All subjects were well informed about the study and potential risk and signed an informed consent before participation. In this retrospective study, a total of 144 patients with CD were enrolled. As reported previously (21–24), the diagnosis of CD was based on the comprehensive analysis of medical history, clinical manifestations, radiological, endoscopic, and histological examinations, as well as laboratory tests. The clinical activity of CD was evaluated based on the Crohn’s Disease Activity Index (CDAI), and the evaluation of endoscopic activity of CD was performed according to the Simple Endoscopic Score for Crohn’s Disease (SES-CD). The gastroenterologists who evaluated endoscopic activity of CD had all been practicing endoscopy for more than 5 years and had previous experience with IBD scores. They all belonged to our clinical center (Sichuan Provincial People’s Hospital) for IBD management and were accustomed to clinical trials that included endoscopic evaluations. The Montreal classification was applied to categorize CD phenotypes. Mucosa biopsies were collected during endoscopic examination. Gender- and age-matched healthy individuals (n = 239) who underwent routine physical examinations in our hospital were enrolled as controls. Clinical characteristics and laboratory parameters of enrolled subjects were retrieved from the electronic medical record database of our hospital. Exclusion criteria were as follows: smoking, excessive drinking, hematopoietic system disease, hepatobiliary disease, coagulation abnormalities, taking medications that can affect blood cell components or serum biochemistry profiles, hypertension, diabetes, other systemic autoimmune diseases, other gastrointestinal diseases, and cancers. The demographics and clinical parameters of CD patients and healthy controls are described in **Table 1**. Anti-TNF therapy and initial response evaluation were performed as previously (22). Briefly, among 144 CD patients, 42 of them were administered with anti-TNF-α mAb (5 mg/kg, Infliximab, IFX) at weeks 0, 2 and 6 for induction. Patients with complete, partial response or primary non-response were defined by the physician’s assessment of symptoms at week 12-14 after initial administration.

**Table 1:** Demographics and clinical parameters of CD patients and healthy controls.

	CD	Healthy controls	*p* value
Number of subjects (n)	144	239	–
Age (year)	37.5 ± 10.5	39.2 ± 11.5	0.1487
Gender (n)			
Female	78	114	0.2462
Male	66	125	
Disease duration (months)	30.5 ± 14.9	–	–
Age at diagnosis (n)			
A1	1	–	–
A2	98	–	–
A3	45	–	–
Disease location (n)			
L1	17	–	–
L2	24	–	–
L3	51	–	–
L4	0	–	–
Disease behavior (n)			
B1	58	–	–
B2	55	–	–
B3	31	–	–
blood NEU (%)	69.22 ± 9.76↑	55.47 ± 8.32	<0.0001
serum ALB (g/L)	35.68 ± 6.58↓	44.73 ± 2.51	<0.0001
serum total BIL (μmol/L)	5.91 ± 2.05 ↓	12.48 ± 5.66	<0.0001
CRP (mg/L)	30.10 ± 17.00	–	–
ESR (mm/hour)	66.43 ± 31.38	–	–
NAR	2.02 ± 0.55 ↑	1.33 ± 0.21	<0.0001
NBR	13.02 ± 4.66↑	6.42 ± 6.44	<0.0001

Data are presented as mean ± SD when applicable.

NEU, neutrophil percentage; CD, crohn’s disease; ESR, erythrocyte sedimentation rate; CRP, C-reactive protein; ALB, albumin; BIL, bilirubin; NAR, neutrophil-to-albumin ratio; NBR, neutrophil-to-bilirubin ratio.

The differences of all parameters between CD patients and healthy donors were examined by Student’s t test (unpaired, two-tailed), except for the gender which was examined by Chi-square test. p < 0.05 was considered statistically significant.

Phenotypes of CD were classified according to the Montreal classification system. ↑ and ↓, increased and decreased compared with healthy controls, respectively.

### Cytokine Measurement

Serum samples were collected using serum separator tube (BD Biosciences), which were sent to the laboratory and processed within 1 hour and stored at −80°C. Cytokine levels in sera were measured by enzyme-linked immunosorbent assay (ELISA) as described previously (21, 22). ELISA kits for TNF-α and IFN-γ were purchased from BioLegend (San Diego, CA, USA).

### RNA Extraction and PCR

As described previously (22, 23), total RNA was extracted from mucosa tissues using TRIzol reagent (Thermo Scientific) and mRNA and microRNA (miR) were subjected to reverse transcription according to the manufacturer’s instructions using 5×All-In-One RT MasterMix kit (Applied Biological Materials Inc., Richmond, British Columbia, Canada) and RT-PCR miRcute miRNA First-Strand cDNA Synthesis Kit (Tiangen Biotech, Beijing, China), respectively. Gene relative expression was determined by qRT-PCR using a SYBR Green real-time PCR system (Invitrogen, CA, USA). The GAPDH and U6 expression levels were employed to normalize the expression of mRNA and miR, respectively.

### Statistical Analysis

The statistical analysis was performed using a Prism software Version 8.4 (Graphpad Software, San Diego, California, USA). Data are presented as mean ± SD when applicable. Except for the gender which was examined by Chi-square test, unpaired Student’s *t* test (two-tailed) was performed to examine the differences of parameters between CD patients and healthy controls, or between IFX responders and primary non-responders. Receiver operator curves (ROC) analysis was performed to assess the performance of each biomarker in discriminating between indicated groups. Correlations between two parameters were examined using Pearson’s correlation analysis. p value < 0.05 was set as statistically significant.

## Results

### Demographics and Clinical Parameters of the Participants

**Figure 1:**
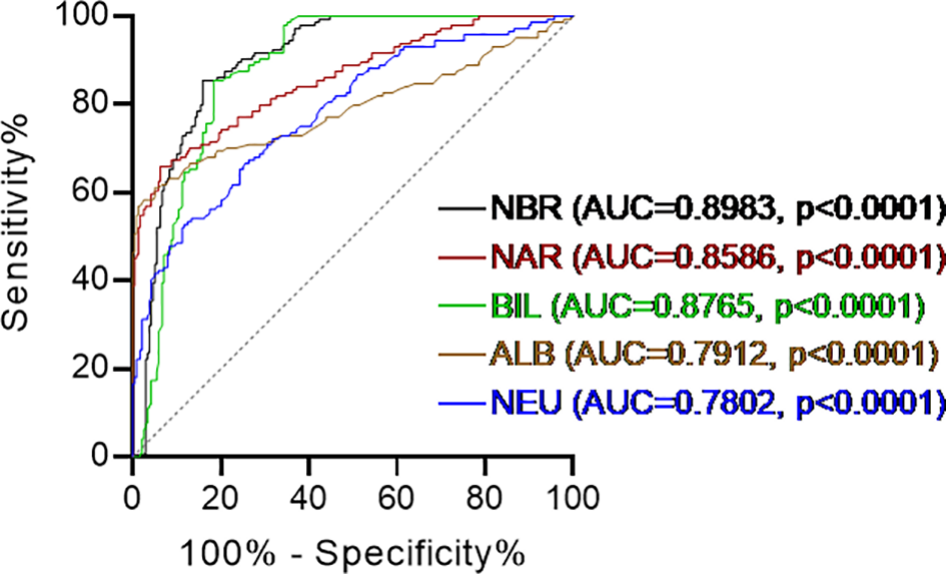
Receiver operating characteristics (ROC) curve analysis. Discriminate abilities of serum levels of albumin (ALB), total bilirubin (BIL), blood neutrophil percentage (NEU) and the neutrophil-to-albumin ratio (NAR), neutrophil-to-bilirubin ratio (NBR) in patients with Crohn’s disease (CD) and healthy controls. Receiver operating characteristics (ROC) curve analysis was performed. AUC, area under the ROC curve. p < 0.05 was considered significant.

As shown detailedly in **Table 1**, we enrolled 144 patients with CD (78 female, 66 male). Their mean age was 37.5 ± 10.5 years old and disease duration was 30.5 ± 14.9 months. We also included 239 healthy individuals (114 female, 125 male) who underwent routine physical examinations in our hospital to serve as controls, whose mean age was 39.2 ± 11.5 years old. CD patients and healthy controls were gender- and age-matched (p=0.1487 and p=0.2462, respectively). Phenotypes of CD were classified according to the Montreal classification system. Based on complete blood cell and serum biochemistry examinations, CD patients showed remarkably higher neutrophil percentage (NEU, 69.22 ± 9.76%, p < 0.0001) and lower serum ALB (35.68 ± 6.58 g/L, p < 0.0001), BIL (5.91 ± 2.05 μmol/L, p < 0.0001) levels compared to healthy controls (NEU, 55.47 ± 8.32%; ALB, 44.73 ± 2.51 g/L; BIL, 12.48 ± 5.66 μmol/L). These findings were consistent with existing studies (20, 25). Next, we combined NEU from complete blood cell tests with ALB and BIL from serum biochemistry examinations. NAR and NBR were calculated as the ratio of NEU-to-ALB (g/L) and NEU-to-total BIL (μmol/L), respectively. Both NAR (2.02 ± 0.55, p<0.0001) and NBR (13.02 ± 4.66, p<0.0001) were significantly increased in patients with CD compared with those in healthy controls (NAR, 1.33 ± 0.21; NBR, 6.42 ± 6.44). Additionally, we performed receiver operating characteristics (ROC) curve analysis and checked the diagnostic accuracy. The area under the ROC curve (AUC) between 0.5 and 0.6 suggests the bad accuracy of a diagnostic test. AUC between 0.6 and 0.7 suggests sufficient accuracy, between 0.7 and 0.8 good accuracy, between 0.8 and 0.9 very good accuracy, whereas AUC higher than 0.9 suggests the excellent accuracy of a diagnostic test (26). Although all these 5 indices (NEU, ALB, BIL, NAR, and NBR) showed significant discriminative abilities between CD patients and healthy controls, NAR (AUC = 0.8586) appeared to be stronger than NEU (AUC = 0.7802) or ALB (AUC =0.7912) alone and NBR (AUC = 0.8983) was stronger than NEU (AUC = 0.7802) or BIL (AUC = 0.8765) alone (**Figure 1**). These data suggest that NAR and NBR could be useful biomarkers for CD diagnosis.

### Associations of NAR and NBR With CD Activity

**Figure 2:**
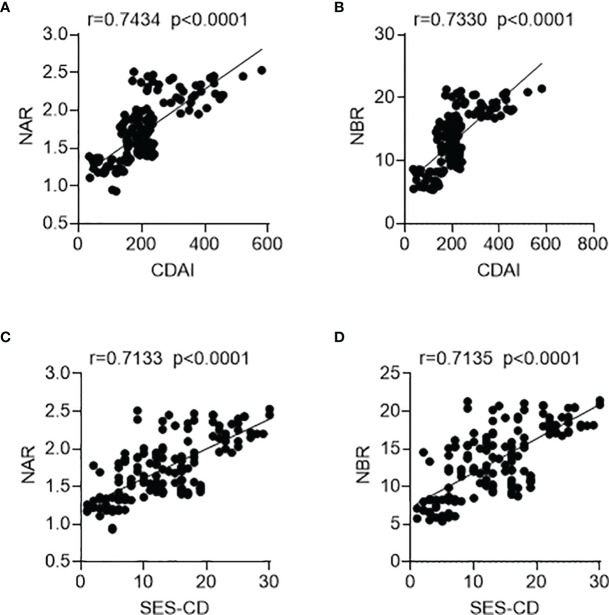
Associations of NAR and NBR with disease activity. A total of 144 patients with CD were enrolled. For clinical activity analysis, **(A)** NAR and **(B)** NBR were positively correlated with CDAI. For mucosal disease activity analysis, **(C)** NAR and **(D)** NBR were also positively associated with SES-CD. The correlation analysis was performed using Pearson’s correlation. p < 0.05 was considered significant.

We next sought to determine whether NAR and NBR could be applied as biomarkers for disease activity. We employed the CDAI for the clinical activity assessment. As shown in **Figures 2A, B**, both NAR and NBR were positively correlated with the clinical activity of CD (NAR, r = 0.7434, p < 0.0001; NBR, r = 0.7330, p < 0.0001). Additionally, since mucosal healing has been thought to indicate a favorable long-term outcome of IBD and thus become a novel therapeutic goal in the disease (4), we further employed the SES-CD to evaluate mucosal disease activity in patients with CD. Similarly, both NAR and NBR were positively correlated with the mucosal disease activity of CD (NAR, r = 0.7133, p < 0.0001; NBR, r = 0.7135, p < 0.0001) (**Figures 2C, D**). Furthermore, we also compared these two indexes with NEU, ALB, and BIL in regarding reflecting CD activity. **Table 2** showed serum ALB and BIL also significantly indicated the disease activity in CD, which were in line with previous evidences (20, 25). Although NEU was markedly increased in CD patients, but itself was not adequate to distinguish the disease activity. Notably, we found that NAR (CDAI, r = 0.7434; SES-CD, r = 0.7133) had a more intimate association with CD activity than ALB alone (CDAI, r = −0.7029; SES-CD, r = −0.6731) and similar results were observed between NBR (CDAI, r = 0.7330; SES-CD, r = 0.7135) and BIL (CDAI, r = −0.6898; SES-CD, r = −0.7014). These data implicate that NAR and NBR might be better biomarkers to reflect disease activity than NEU, ALB or BIL alone.

**Table 2:** Associations of different parameters with CD activity.

	CDAI		SES-CD	
	r	p	r	p
NAR	0.7434	<0.0001	0.7133	<0.0001
NBR	0.7330	<0.0001	0.7135	<0.0001
serum ALB (g/L)	-0.7029	<0.0001	-0.6731	<0.0001
serum BIL (μmol/L)	-0.6898	<0.0001	-0.7014	<0.0001
blood NEU (%)	0.2620	0.0016	0.2335	0.0545

CDAI, Crohn’s Disease Activity Index; SES-CD, Simple Endoscopic Score for Crohn’s Disease.

Associations of different parameters with the disease activity in CD patients (n = 144) were analyzed by Pearson’s correlation. p < 0.05 was considered statistically significant.

### Associations of NAR and NBR With Inflammatory Load in CD Patients

To further verify the biomarker performance of NAR/NBR for the disease activity, we explored the associations between these two indexes and inflammatory indices in CD patients (**Table 3**). First of all, we look at serum inflammatory factors. CRP, as well as ESR, has been well-reported as disease activity biomarkers of IBD, and we found NAR and NBR were positively correlated with CRP and ESR. Additionally, increased levels of serum pro-inflammatory cytokines are a hallmark of IBD such as TNF-α and IFN-γ, both of which were also significantly correlated with NAR and NBR. Next, we analyzed mucosa expression of TNF-α and IFN-γ and found they were also positively associated with NAR and NBR in CD patients. Particularly, our previous studies have provided compelling evidences to support the role of mucosal miR-301a as a biomarker for diagnosis and disease activity in both CD and UC (22, 23). Here, we employed mucosa expression of miR-301a as an inflammatory indice of CD patients, which was also positively associated with NAR and NBR. These observations indicate that NAR and NBR might help to monitor the systemic and mucosal inflammatory load in CD patients.

**Table 3:** Associations of NAR and NBR with inflammatory load in CD patients.

	NAR		NBR	
	r	p	r	p
CRP (mg/L)	0.5728	<0.0001	0.7099	<0.0001
ESR (mm/hour)	0.6160	<0.0001	0.7509	<0.0001
serum TNF-α (pg/mL)	0.6241	<0.0001	0.7534	<0.0001
serum IFN-γ (pg/mL)	0.5893	<0.0001	0.6152	<0.0001
Mucosal TNF-α mRNA expression	0.5142	<0.0001	0.6086	<0.0001
Mucosal IFN-γ mRNA expression	0.5796	<0.0001	0.6829	<0.0001
Mucosal miR-301a mRNA expression	0.5740	<0.0001	0.5880	<0.0001

Associations of NAR and NBR with inflammatory indices in CD patients (n = 144) were analyzed by Pearson’s correlation. p < 0.05 was considered statistically significant.

The expression levels of mucosal mRNA shown above are relative values compared to those from healthy controls (n=56).

### NAR and NBR Predict Response to IFX in CD Patients

**Figure 3:**
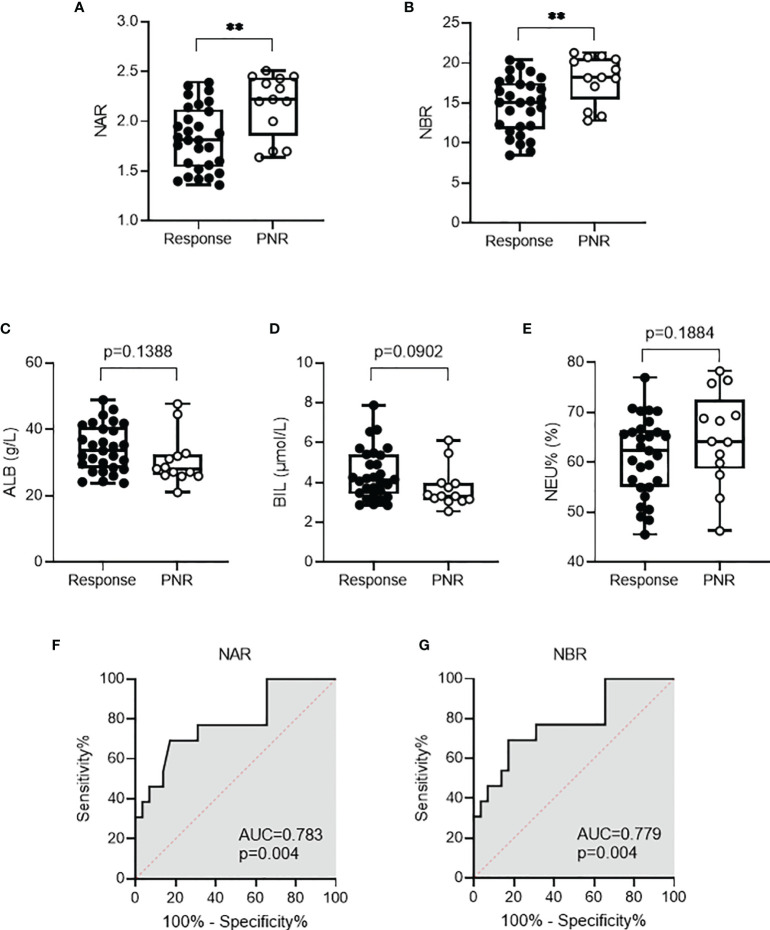
NAR and NBR predict response to infliximab (IFX) in CD patients. CD patients (n = 42) received IFX induction therapy including 29 with complete or partial response and 13 with primary non-response. Differences of **(A)** NAR, **(B)** NBR, **(C)** ALB, **(D)** BIL, and **(E)** NEU were determined between patients with initial response to IFX and those who had primary non-response. **p < 0.01, Student’s t test (unpaired, two-tailed) was performed. Abilities of **(F)** NAR and **(G)** NBR to discriminate IFX responders from primary non-responders were determined by ROC curve analysis was performed. p < 0.05 was considered significant.

Because of the application of biologics (such as anti-TNF agents), the therapy for IBD has been revolutionized, therefore remarkably improving the quality of life. However, approximately one-third of patients with IBD fail to or limitedly respond to IFX induction therapy. Finding optimal predictors of initial response to IFX has been emphasized to selectively treat patients who have the highest chance of responding. Thus, we asked whether NAR and NBR could be potential predictive biomarkers in this scenario. Among 144 enrolled CD patients, 42 received IFX induction therapy, including 29 with complete or partial response and 13 with primary non-response. As shown in **Figures 3A, B**, patients with initial response to IFX showed significantly lower NAR (1.83 ± 0.32, p=0.0026) and NBR (14.68 ± 3.44, p=0.0042) than those who had primary non-response (NAR, 2.17 ± 0.31; NBR, 18.02 ± 2.96). However, we did not observe any significant differences between initial responders and primary non-responders in regard to serum ALB, BIL levels and blood NEU (**Figures 3C–E**). Next, we performed the receiver operating characteristics (ROC) curve analysis to determine the discriminative abilities of NAR and NBR as predictors for response to IFX induction. NAR (AUC = 0.783, p = 0.004), as well as NBR (AUC = 0.779, p = 0.004) (**Figures 3F, G**), significantly discriminated between initial responders and primary non-responders to IFX induction therapy.

## Discussion

With the progress of IBD management, biomarkers are utilized not only for diagnosis and monitoring but also for individualized therapies. An ideal biomarker should have the following features and advantages: non-invasive, high sensitivity and specificity, easy to implement, and affordable (27). To date, no biomarkers that meet these requirements have been found to be alternatives to endoscopy. Such as anti-saccharomyces cerevisiae antibodies (ASCA), they have been suggested as stable markers in the management of CD (28), but the high prevalence of ASCA is also found in coeliac disease (29, 30). Except CRP and fecal calprotectin, most biomarkers need to be validated in large populations. Given the application of appropriate biomarkers reduces the frequency of expensive and invasive endoscopy, which leads to a lower healthcare burden on patients, new biomarkers for the management of IBD are being strongly expected.

In the current study, we analyzed the alterations of two novel biomarkers derived from blood NEU in combination of serum ALB and BIL in CD patients, demonstrating that: 1) NAR and NBR were significantly increased in patients with CD compared to those in healthy controls; 2) both indexes showed significantly positive correlations with CD activity and inflammatory load (both systemic and mucosal); 3) both NAR and NBR discriminated CD patients who completely or partially responded to IFX induction therapy from those with primary non-response. These observations prompt us to speculate their potential to serve as predictive biomarkers in the diagnosis and treatment of CD.

It is critical to timely assess the disease activity in patients with IBD in order to select optimal therapeutic strategies and have better prognosis. Endoscopic biopsy is the gold standard for evaluating IBD inflammatory activity, especially monitoring mucosal healing (31). However, endoscopy may lead to injury during the operation and is sometimes even a contraindication to severe IBD. In terms of noninvasive biomarkers, many predictive factors have been reported to reflect the disease activity. For example, conventional serum parameters such as CPR and ESR are the most widely employed in clinic as indicators for inflammatory activity. However, the poor specificity largely limits their clinical significance since CPR and ESR also rise fast under the situations of tissue necrosis, infection, or other causes (32, 33). Thus, it is insufficient to employ them alone but as supplements to endoscopy or other approaches for the disease activity assessment (27). In addition, fecal calprotectin is now one of the greatest biological indicators for discriminating IBD and assessing disease activity. Although it can be utilized in clinical practice, it is not frequently employed because of the high cost, long time requirement, and difficulties in sample collection and processing (34). As a result, exploration of simpler, more accessible/efficient biomarkers is urgently required.

Of note, novel indexes derived from combinations of two blood cell parameters have emerged as potent tools to predict IBD activity. Recently, Wang et al. conducted a meta-analysis including 2185 IBD patients and 993 healthy controls. In this study, blood NLR were found to be significantly increased in patients with IBD compared with that in healthy controls and more importantly, patients with active IBD showed higher NLR than those in remission, suggesting a role of NLR as a valuable biomarker to predict IBD activity (35). Higher PLR and lower LMR were found between patients with CD and control subjects (17, 18). Interestingly, informative parameters combining blood cell counts and serum biochemical indices such as NAR, which is a newly revealed index indicating systemic inflammation and it has been used in inflammatory, vascular diseases, and cancers (36–38). In patients with cardiogenic shock, NAR is observed to be a more sensitive diagnostic marker than blood neutrophil or serum albumin level alone (36). It has been well-documented that IBD patients have lower levels of serum ALB and hypoproteinemia might reduce therapeutic efficiencies of biologics (10). However, to our best knowledge, there are no studies using NAR in the IBD area. In this study, although ALB alone could be a sensitive diagnostic marker in CD, NAR show a higher application value to reflect the CD activity and systemic inflammatory load.

In addition to NAR, we revealed a novel index as the NEU-to-BIL ratio, which had not yet been defined. Conventionally, BIL is considered as an end product of heme degradation and it is cytotoxic at high concentrations. Elevated serum BIL levels can be a consequence of heme overproduction from hemoglobin caused by hemolysis or impaired hepatic conjugating activity. Recent investigations have demonstrated a variety of biological properties of this the metabolite of iron porphyrin at physiological concentrations, such as anti-oxidative and anti-inflammatory (39, 40). In animal models of experimental colitis, BIL protects mice from DSS-induced colitis, possibly *via* reducing mucosal leukocyte infiltration, maintaining redox homeostasis (41), and promoting BIL reabsorption *via* enterohepatic cycling (42). With regard to human IBD, patients with the Gilbert’s polymorphism have a decreased risk of CD (43) and patients with IBD show significantly lower levels of BIL than controls (20). Decreased BIL levels indicates higher CRP, ESR, fecal calprotectin and disease activity scores (20). Here, we also find a decrease in the serum levels of BIL in CD patients. More notably, our findings show, for the first time, that CD patients display significant higher NBR, which are more sensitive to indicate the associations with the CDAI, SES-CD, CRP, ESR, and inflammatory marker expression (TNF-α, IFN-γ, miR-301a) than BIL or NEU alone.

The application of anti-TNF agents has been a cornerstone in the therapy for IBD, which changes the natural history of both CD and UC. IFX, as the most-investigated anti-TNF agent, is effective for induction and maintenance of clinical remission and mucosal healing in IBD patients. Accumulating evidences demonstrate that trough concentration of IFX could guide and optimize therapeutic strategies (44). Unfortunately, one-third of IBD patients fail to initially respond to IFX and have no or limited clinical benefit after the induction therapy (12-14 weeks post induction), which is defined as “primary non-response” (44). Given the fact that IFX does not work in all patients and has possibilities of major adverse effects as well as high expense, it is crucial to identify biomarkers to predict primary non-response to IFX in IBD patients. Several predictive factors of IFX response in IBD patients have been revealed, such as patient-related factors (age, weight, smoke, et al.) and disease-related factors (disease duration, CRP, ALB, et al.) (10). However, most of them have not demonstrated utility, and many others remain controversial. In terms of ALB, several studies suggest that low serum albumin levels are consistently associated with reduced response rates in UC patients with IFX treatment, which is also reflected in hypoalbuminaemic patients, who had lower infliximab serum levels than control subjects (45, 46). As for CD patients, little is known about the predictive efficacy of ALB in response to IFX. In our current study, we found NAR and NBR displayed significant capacities to discriminate IFX responders from primary non-responders. ALB, BIL, or NEU% alone did not show evident differences between two groups.

In summary, we here employed two novel indexes NAR and NBR, which were significantly increased in CD patients and could be sensitive biomarkers for the disease activity and inflammatory load. Importantly, NAR and NBR showed potential values to predict response to IFX induction therapy in CD patients. We realize that several limitations need to be further addressed: our current observations should be validated in future large sample clinical studies, which might involve UC patients; follow-up studies are required to confirm the predictive values of NAR and NBR in CD; whether these indexes could be used to predict secondary non-response to IFX or other biologics (such as Vedolizumab and Ustekinumab). Nevertheless, our findings provide evidences to apply NAR and NBR, which may be better than ALB, BIL, or NEU alone, in the diagnosis, activity monitoring, and IFX response prediction in patients with CD.

## Data Availability Statement

The original contributions presented in the study are included in the article/supplementary material. Further inquiries can be directed to the corresponding authors.

## Ethics Statement

The studies involving human participants were reviewed and approved by the Institutional Review Board for Clinical Research of Sichuan Provincial People’s Hospital (No.201685, 2020204). The patients/participants provided their written informed consent to participate in this study.

## Author Contributions

CH and CG conceptualized and designed the study plan and edited the manuscript. ZZ, YZ, and XY collected clinical information and samples from enrolled subjects. LL, YP, and CG diagnosed the patients. CH, CG, and ZZ analyzed the data and prepare the original draft. All authors discussed and revised the manuscript, and agreed to the published version of the manuscript.

## Funding

This work is financially supported by grants from the National Natural Science Foundation of China (82070985, 82170579) and Foundation of Sichuan Science and Technology Department (2021JDJQ0044).

## Conflict of Interest

The authors declare that the research was conducted in the absence of any commercial or financial relationships that could be construed as a potential conflict of interest.

## Publisher’s Note

All claims expressed in this article are solely those of the authors and do not necessarily represent those of their affiliated organizations, or those of the publisher, the editors and the reviewers. Any product that may be evaluated in this article, or claim that may be made by its manufacturer, is not guaranteed or endorsed by the publisher.

## Abbreviations

IBD, inflammatory bowel disease; CD, Crohn’s disease; UC, ulcerative colitis; ALB, albumin; BIL, bilirubin; NAR, neutrophil-to-albumin ratio; NBR, neutrophil-to-bilirubin ratio; PLR, platelet-to-lymphocyte ratio; NLR, neutrophil-to-lymphocyte ratio; LMR, lymphocyte-to-monocyte ratio; ROC, receiver operating characteristics; PCR, polymerase chain reaction; qRT-PCR, quantitative Real-time PCR; ELISA, enzyme-linked immunosorbent assay; IFX, infliximab; ESR, erythrocyte sedimentation rate; CRP, C-reactive protein; IL, interleukin; TNF, tumor necrosis factor; IFN, interferon; miR, microRNA; CDAI, Crohn’s Disease Activity Index; SES-CD, Simple Endoscopic Score for Crohn’s Disease; AUC, area under the curve.
